# Imbalanced Regional Development of Acute Ischemic Stroke Care in Emergency Departments in China

**DOI:** 10.1155/2019/3747910

**Published:** 2019-08-06

**Authors:** Jianguo Li, Jingming Liu, Yuefeng Ma, Peng Peng, Xiaojun He, Wei Guo

**Affiliations:** ^1^Emergency Department, Beijing Tiantan Hospital, Capital Medical University, Beijing 100070, China; ^2^Department of Chinese Journal of Emergency Medicine, The Second Hospital of Zhejiang University Medical College, Hangzhou 310009, China; ^3^The First Affiliated Hospital of Xinjiang Medical University, Wulumuqi 830001, China

## Abstract

**Objective:**

Most patients of acute ischemic stroke (AIS) receive treatments in the department of emergency in China. We aimed to examine the status of AIS diagnosis and treatment and the impact of green pathway operation in different regions of China.

**Methods:**

In this nationwide survey, information regarding the emergency care of AIS was collected from 451 hospitals in different regions of China, by interviewing 484 physicians from these hospitals. Structured questionnaire was used to explore the status of AIS care and impact of the green pathway.

**Results:**

445 hospitals from 18 provinces, 4 municipalities, and 3 ethnic autonomous regions in China were included in the present study. Overall, the proportion of door-to-needle time (DNT) less than 60 min was 66.08% in the enrolled hospitals (*n* = 298). Stratified by regions, the results suggested that hospitals located in East regions had shorter DNT time (*P*=0.036), and more proportion of rtPA (*P* < 0.001) than those in West regions. Further analysis suggested that hospitals with a green channel were more likely to shorten DNT and improve the proportion of rtPA (*P* < 0.01).

**Conclusion:**

Considerable regional differences were observed in terms of DNT time and thrombolysis rates in the departments of emergency in China. Further studies are required to confirm the regional differences in AIS care in China.

## 1. Introduction

Approximately 15 million people suffered from stroke attack every year globally, with about 5 million deaths [1]. It is estimated that >7 million Chinese have strokes, and approximately 80% of them are acute ischemic stroke (AIS) by Chinese National Stroke Registry (CNSR) [2]. Rising incidence and morbidity of ischemic stroke have created a heavy economic burden to the Chinese healthcare system [3, 4].

Thrombolysis with recombinant tissue-type plasminogen activator (rtPA) is the only FDA-approved therapy for AIS [5–7] and recommended by most guidelines [8, 9]. However, it has a narrow therapeutic time window of 3 to 4.5 hours after AIS onset [10]. With the reported rate of using rtPA between 4 and 10% in the United States [11–13] and only 2% in China [2], many barriers of giving rtPA have been identified, including prehospital or in-hospital delay, short treatment time window, and lack of hospital infrastructure.

Recently, there have been several efforts to reduce in-hospital time delays, including reorganization of the Emergency Department (ED) [14], use of point-of-care international normalized ratio testing [15], acute stroke pathway [16], and establishment of green pathways for stroke thrombolysis [17] in China. However, the lack of effective supervision and continuous optimization of work flow remain the major obstacles to make significant breakthroughs in reducing in-hospital delays.

Given that most patients of stroke receive treatments in the department of emergency in China, more efforts need to be made to study the status of AIS diagnosis and treatment in emergency rooms in China, as to find out the factors related to the time required for rtPA, with a target benchmark DNT for rtPA administration less than 60 min [18, 19]. For this purpose, the present study aims to examine the current status of management of AIS in emergency departments through an online questionnaire in different regions of China.

## 2. Materials and Methods

### 2.1. Study Design

In this nationwide survey, information regarding the emergency care of AIS was collected from 451 hospitals in different regions of China, by interviewing 484 physicians from these hospitals. These physicians were selected from the hospitals registered by the National Emergency Medical Association and asked to complete a questionnaire online. The areas are divided into three major blocks, including East, Middle, and West regions, which are mainly based on the geographical location and economic development levels. The East region includes 11 provinces, municipalities, or autonomous regions, namely, Liaoning, Beijing, Tianjin, Shandong, Jiangsu, Shanghai, Zhejiang, Fujian, Guangdong, Guangxi, and Hainan. The Middle region includes Shanxi, Inner Mongolia, Jilin, Heilongjiang, Anhui, Jiangxi, Henan, Hubei, and Hunan. The West region refers to Shanxi, Gansu, Qinghai, Ningxia, Xinjiang, Sichuan, Chongqing, Yunnan, Guizhou, and Tibet. In particular, these physicians were subjected to an online questionnaire with 19 items including AIS emergency treatment, referral, proportion of rtPA, and so on (Supplementary Materials 1). The study was approved by the Ethics Committee of Beijing Tiantan Hospital, and the need for informed consent was waived.

### 2.2. Green Pathway

By integrating various treatment pathways in the department of emergency, neurology, neurosurgery, pharmacy, laboratory, and radiology, a green pathway for the management of AIS patients was established in some Chinese hospitals. Briefly, the ED physicians are expected to examine the suspected stroke patients immediately as the patients arrived at ED. When a patient had at least 1 warning sign of AIS, the ED physician should activate the green pathway by stamping with the seal of “Green Pathway.” Once the prescription is received, the pharmacists make it a priority to dispense the medicines promptly and the radiologists make the CT scanner ready for the patient. Consequently, the green pathway saved a considerable amount of valuable time that would have been spent in payments, obtaining medicines, waiting to be examined, and waiting for results.

### 2.3. Statistical Analysis

Proportions were used for descriptive analysis. Comparisons between two groups were made using Student's tests or chi-square tests or Fisher's exact tests. For all analysis, a 2-tailed probability value of 0.05 was considered significant and analyses were performed using R 3.1.0.

## 3. Results

### 3.1. Characteristics of Enrolled Hospitals

**Figure 1:**
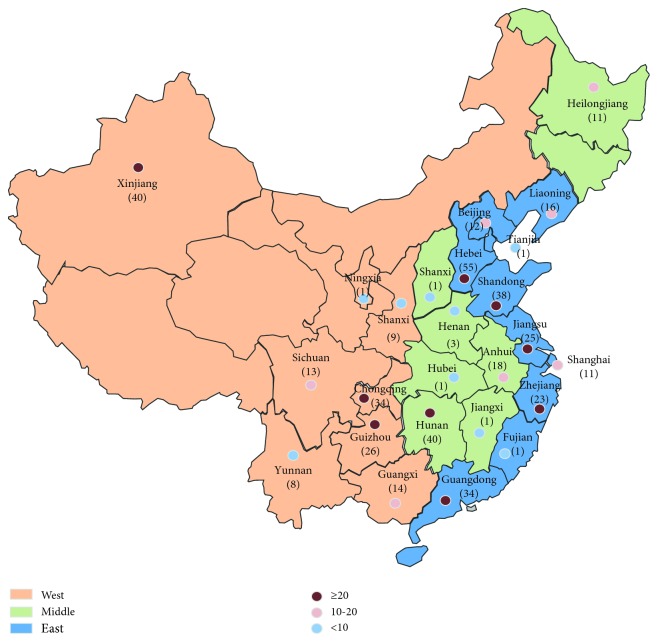
Map of regions involved in the survey.

Apart from 6 hospitals with missing data, a total of 445 hospitals from 18 provinces, 4 municipalities, and 3 ethnic autonomous regions in China were included in the present study. Among these hospitals, 55, 40, 40, 38, 34, 34, 26, 25, and 23 of them are located in Hebei (12.36%), Hunan (8.99%), Xinjiang (8.99%), Shandong (8.54%), Chongqing (7.64%), Guangdong (7.64%), Guizhou (5.84%), Jiangsu (5.62%), and Zhejiang (5.17%), respectively, with the numbers of hospitals above 20. Nine regions with numbers of included hospitals under 10 were Fujian (0.22%), Hubei (0.22%), Jiangxi (0.22%), Ningxia (0.22%), Tianjin (0.22%), Shanxi (0.22%), Yunnan (1.80%), Henan (0.66%), and Shaanxi (1.98%) (Figure 1).

As shown in Table 1, 63.41% of the hospitals were Grade Three hospitals. 74.72% had more than 7 physicians, and 55.43% of the hospitals had a daily medical attendance more than 500 patients in the department of emergency. Of the interviewed 484 physicians from these 451 hospitals, 140 (28.9%) were chief physicians and 208 (42.9%) were associate chief physicians.

### 3.2. Regional Difference of AIS Treatment

During the data collection period, there are 217 hospitals with the highest proportion (48.12%) that the onset-to-needle time (ONT) was less than 4.5 hours. The proportion of DNT less than 60 min was 66.08% (*n* = 298), while the DNT more than 60 min was 33.92%.

There are 51.69%, 18.88%, and 29.44% of hospitals located in the East, Middle, and West regions of China, respectively. Stratified by regions, the results suggested that hospitals located in East regions had shorter DNT time (*P*=0.036) and more proportion of rtPA (*P* < 0.001) than those in West regions (Table 2). In addition, the proportion of hospitals with green pathway was highest in East regions (90%), while the proportion was lower in Middle and West (77.38% and 85.50%, *P*=0.015).

### 3.3. Impact of Green Channel on AIS Treatment

In the present study, up to 86.03% (*n* = 388) of hospitals have established the green channel. To further study the impact of green channel on stroke treatment, we analyzed the information according to with or without green channel. As shown in Table 3, hospitals with the green channel were more likely to have shorten DNT time (*P* < 0.001) and improve the proportion of rtPA treatment (*P* < 0.001, Table 3).

### 3.4. Impact of Green Channel on AIS Treatment in Different Blocks

To further study whether the impact of green channel on stroke treatment is different among the three blocks (East, Middle, and West), we next carried out the subgroup analysis. As shown in Table 4, we found that the DNT time is shorter in hospitals with green pathway in any area, but the difference is more pronounced in the Middle (East: *P*=0.071; Middle: *P*=0.002; West: *P*=0.680). Besides, the influence of green pathway on the proportion of rtPA is similar in different blocks, with significance in East and Middle areas (East: *P* < 0.001; Middle: *P* < 0.001; West: *P*=0.105).

## 4. Discussion

In the present study, we provide a comprehensive map of the current status of AIS treatment in emergency departments across China by using an online questionnaire, revealing large regional differences in AIS care in China. We also presented that implementation of green pathway greatly improved the time from arrival to thrombolysis, which eliminated one of the important barriers to eligibility for rtPA. In our analysis, implementation of green pathway could significantly increase the rate of rtPA by reducing in-hospital delay.

A patient with typical ischemic stroke loses nearly 1.9 million neuronal cells per minute delay of thrombolysis, which highlights the importance of a quick therapeutic approach during AIS [20]. Early reports of rtPA in routine clinical practice have demonstrated average DNTs being reduced from 100 to 48 minutes, with improvement over time suggesting a learning curve [21–23]. Despite this, only a 1.5% thrombolysis rate was observed for all AIS patients during the period of 2006 to 2008 conducted by a multihospital survey in Taiwan [24]. Recently, great successes have been achieved through multiple approaches like the reorganization of the emergency room [14], the introduction of an acute stroke team [25], a stroke code protocol [26], and green pathway. Prior to use of the green pathway, patients had to wait for the diagnosis by neurologists, the approvement of CT request, and so on. The green pathway eliminated many of those steps by strengthening the cooperation of emergency, clinical laboratory, and stroke team so that suspected stroke patients could receive treatment quickly. In our study, green pathway usage in hospitals significantly shortens DNT, with 13.14% and 56.44% of hospitals reaching the DNT goal of ≤30 and ≤60 min, respectively. Green channel is not applied for AIS only in China. As acute myocardial infarction (AMI) is a severe type of heart disease with golden hour similar with ischemic stroke, many hospitals have established the green channel for AMI to achieve timely thrombolysis within golden hours in China.

Considerable regional differences were observed in terms of DNT time and thrombolysis rates. However, it could not be explained by the regional differences in scales of hospitals, as no differences were observed regarding the daily medical attendance and numbers of physicians in the emergency departments of included hospitals from different areas of China. Besides, the hospitals included for the survey were all registered public hospitals in the National Emergency Medical Association of China. Therefore, the levels, the mass, and the operation model of these hospitals were roughly similar to each other. Interestingly, more hospitals in the east area of China have established the green pathway, which might improve the quality of AIS care significantly. In addition, lacking the knowledge of AIS treatment and thrombolysis approach, the poor economic condition and poor transportation facilities may contribute to the low rate of rtPA in the West of China.

Our study has several limitations. First, we do not have comprehensive data dividing the reason of those AIS patients who were not treated with rtPA from in-hospital or prehospital delays. A recent review revealed that about half of stroke patients have arrived hospitals within 4 hours over the last 10 years [27]. Second, we do not know whether the green pathway actually shortened DNT. Lacking a control group, our data do not provide answers to this question. Third, the present survey was conducted based on the mobile and social network based-interview, but not direct raw data of AIS care from the enrolled hospitals. In addition, analysis bias might also be induced because of the imbalance of included hospitals.

## 5. Conclusion

Our study provided evidence in real-world practice of AIS care in the emergency departments of China and provided new insights into the association between regional differences, economic development, and medical quality in China. Further studies are required to confirm the roles of green pathway and regional differences in AIS care in China.

## Figures and Tables

**Table 1:** Characteristics of the investigated physicians and enrolled hospitals.

Characteristics	*N*	%
*Profession*		
Chief physician	140	28.9
Associate chief physician	208	42.9
Visiting doctor	114	23.5
Others	23	4.7

*Level*		
Third level	286	63.41
Second level	162	35.92
Other	3	0.67

*Daily medical attendance*		
<500	201	44.57
≥500	250	55.43

*Numbers of physicians*		
<7	114	25.28
7–12	106	23.50
12–21	113	25.06
≥21	118	26.16

*Proportion of AIS (%)*		
<20	300	66.52
≥20	151	33.48

*Door-to-needle time (min)*		
<30	55	12.20
30–60	243	53.88
≥60	153	33.92

*Onset-to-needle time (hours)*		
<4.5	217	48.12
4.5–6.0	118	26.16
≥6	116	25.72

*Proportion of rtPA (%)*		
<10	161	35.70
10–30	29	6.43
30–50	33	7.32
≥50	228	50.55

**Table 2:** Comparison of characteristics in different regions.

Characteristics	Regions			*P* value
	East (*N*=230)	Middle (*N*=84)	West (*N*=131)	
*Daily medical attendance*				
<500	99 (43.04)	40 (47.62)	58 (44.27)	0.350
≥500	131 (56.96)	44 (52.38)	73 (55.73)	

*Numbers of physicians*				
<7	54 (23.48)	22 (26.19)	37 (28.24)	0.602
7–12	51 (22.17)	20 (23.81)	35 (26.72)	
12–21	61 (26.52)	18 (21.43)	33 (25.19)	
≥21	64 (27.83)	24 (28.57)	26 (19.85)	

*Profession*				
Chief physician	84 (36.52)	16 (19.05)	31 (23.66)	0.429
Associate chief physician	99 (43.04)	38 (45.24)	62 (47.33)	
Visiting doctor	45 (19.57)	23 (27.38)	31 (23.66)	
Resident doctor	0 (0)	2 (2.38)	1 (0.76)	

*Green pathway*				
Yes	207 (90.00)	65 (77.38)	112 (85.50)	0.015
No	23 (10.00)	19 (22.62)	19 (14.50)	

*Proportion of IS (%)*				
<20	148 (64.35)	50 (59.52)	97 (74.05)	0.060
≥20	82 (35.65)	34 (40.48)	34 (25.95)	

*Door-to-needle time (min)*				
<30	27 (11.74)	13 (15.48)	15 (11.45)	0.036
30–60	138 (60)	42 (50)	59 (45.04)	
≥60	65 (28.26)	29 (34.52)	57 (43.51)	

*Onset-to-needle time (hours)*				
<4.5	123 (53.48)	32 (38.1)	59 (45.04)	0.151
4.5–6.0	55 (23.91)	25 (29.76)	37 (28.24)	
≥6	52 (22.61)	27 (32.14)	35 (26.72)	

*Proportion of rtPA (%)*				
<10	58 (25.22)	33 (39.29)	66 (50.38)	<0.001
10–30	14 (6.09)	3 (3.57)	12 (9.16)	
30–50	16 (6.96)	8 (9.52)	8 (6.11)	
≥50	142 (61.74)	40 (47.62)	45 (34.35)	

**Table 3:** Effect of green pathway on the treatment of AIS patients.

Characteristics	Green channel		*P* value
	Yes (*N* = 388)	No (*N* = 63)	
*Proportion of IS (%)*			
<20	259 (66.75)	41 (65.08)	0.907
≥20	129 (33.25)	22 (34.92)	

*Door-to-needle time (min)*			
<30	51 (13.14)	4 (6.35)	<0.001
30–60	219 (56.44)	24 (38.1)	
≥60	118 (30.41)	35 (55.56)	

*Onset-to-needle time (hours)*			
<4.5	190 (48.97)	27 (42.86)	0.479
4.5–6.0	102 (26.29)	16 (25.4)	
≥6	96 (24.74)	20 (31.75)	

*Proportion of rtPA (%)*			
<10	116 (29.9)	45 (71.43)	<0.001
10–30	27 (6.96)	2 (3.17)	
30–50	30 (7.73)	3 (4.76)	
≥50	215 (55.41)	13 (20.63)	

**Table 4:** Comparison of AIS treatment with/without green pathway in different regions.

Characteristics	Regions									
	East (*N* = 230)			Middle (*N* = 84)			West (*N* = 131)		
	With GP (*N* = 207)	Without GP (*N* = 23)	*P*	With GP (*N* = 65)	Without GP (*N* = 19)	*P*	With GP (*N* = 112)	Without GP (*N* = 19)	*P*
*Proportion of IS (%)*									
<20	132 (57.39)	16 (69.57)	0.748	40 (61.54)	10 (52.63)	0.667	83 (74.11)	14 (73.68)	1
≥20	75 (32.61)	7 (30.43)		25 (38.46)	9 (47.37)		29 (25.89)	5 (26.32)	

*Door-to-needle time (min)*									
<30	26 (11.3)	1 (4.35)	0.071	12 (18.46)	1 (5.26)	0.002	13 (11.61)	2 (10.53)	0.680
30–60	127 (55.22)	11 (47.83)		37 (56.92)	5 (26.32)		52 (46.43)	7 (36.84)	
≥60	54 (23.48)	11 (47.83)		16 (24.62)	13 (68.42)		47 (41.96)	10 (52.63)	

*Onset-to-needle time (hours)*									
<4.5	112 (48.7)	11 (47.83)	0.735	27 (41.54)	5 (26.32)	0.094	50 (44.64)	9 (47.37)	0.972
4.5–6.0	48 (20.87)	7 (30.43)		21 (32.31)	4 (21.05)		32 (28.57)	5 (26.32)	
≥6	47 (20.43)	5 (21.74)		17 (26.15)	10 (52.63)		30 (26.79)	5 (26.32)	

*Proportion of rtPA (%)*									
<10	44 (19.13)	14 (60.87)	<0.001	18 (27.69)	15 (78.95)	<0.001	52 (46.43)	14 (73.68)	0.105
10–30	14 (6.09)	0 (0)		3 (4.62)	0 (0)		10 (8.93)	2 (10.53)	
30–50	14 (6.09)	2 (8.7)		8 (12.31)	0 (0)		7 (6.25)	1 (5.26)	
≥50	135 (58.7)	7 (30.43)		36 (55.38)	4 (21.05)		43 (38.39)	2 (10.53)	

## Data Availability

The data used to support the findings of this study are included within the article.

## Supplementary Materials

Supplementary Materials Questionnaire for the emergency care of AIS in China.
