# Corrigendum to “Imbalanced Regional Development of Acute Ischemic Stroke Care in Emergency Departments in China”

**DOI:** 10.1155/2019/9752671

**Published:** 2019-11-21

**Authors:** Jianguo Li, Jingming Liu, Yuefeng Ma, Peng Peng, Xiaojun He, Wei Guo

**Affiliations:** ^1^Emergency Department, Beijing Tiantan Hospital, Capital Medical University, Beijing 100070, China; ^2^Department of Chinese Journal of Emergency Medicine, The Second Hospital of Zhejiang University Medical College, Hangzhou 310009, China; ^3^The First Affiliated Hospital of Xinjiang Medical University, Wulumuqi 830001, China

In the article titled “Imbalanced Regional Development of Acute Ischemic Stroke Care in Emergency Departments in China” [1], there was an error in Figure 1 where the Taiwan Island was missing. The corrected version of Figure 1 is shown as follows.

## Figures and Tables

**Figure 1:**
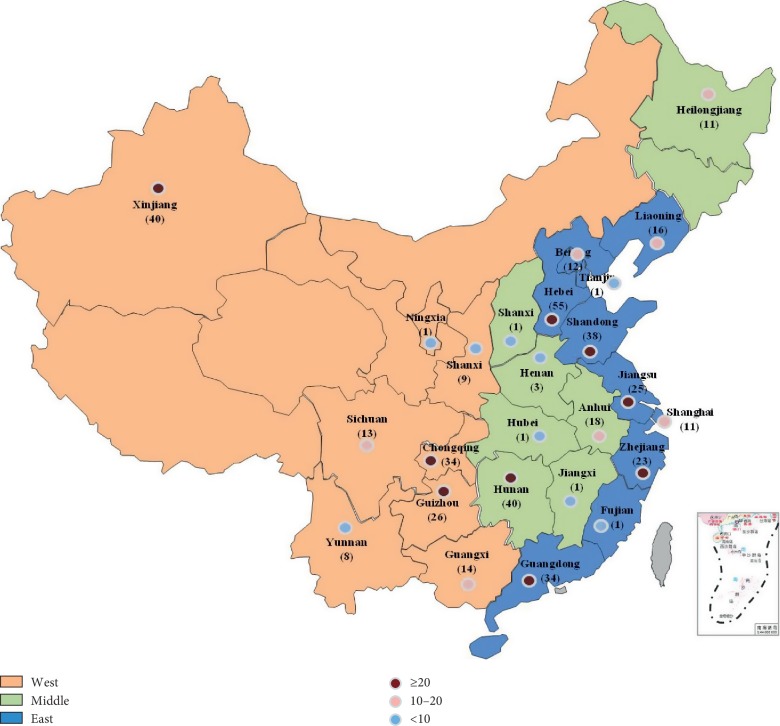
Map of regions involved in the survey.
