# Supplementary material for: Imbalanced Regional Development of Acute Ischemic Stroke Care in Emergency Departments in China
Source: Emerg Med Int. 2019 Aug 6;2019:3747910. doi: 10.1155/2019/3747910 (PMC6701302; doi:10.1155/2019/3747910)
Supplement: Supplementary Materials — Questionnaire for the emergency care of AIS in China. [file 3747910.f1.docx]

**Imbalanced regional development of acute ischemic stroke care in emergency departments in China**

**Supplementary Material**

Supplementary 1: Questionnaire for the emergency care of AIS in China

1. Which level is your hospital? A) 3A B) 3B C) 2A D) others
2. Generally, how many patients are treated in the department of Emergency of your hospital? A) <300 B) 301-500 C) 501-700 D)701-900 E)901-1200 F) >1200
3. The percentage of AIS in all patients with stroke is about: A)<5% B)6-10% C)11-20% D) 21-30% E)31-40% F)>40%
4. When arrived, the time from onset of AIS is about: A)<3h B)3.1-4.5h C)4.6-6h D)>6h
5. Is thrombolysis performed in your hospital? A) Yes B) No
6. Is Green Channel established in your hospital? A) Yes B) No
7. For AIS, which approach was available in your hospital? A) Embolectomy B) Intracranial stenting C) Carotid artery stenting D) carotid endarterectomy E) others
8. Which department performs thrombolysis in your hospital? A) Emergency B) Neurology C) Neurosurgery D) ICU E) others
9. Which place is thrombolysis performed? A) Emergency B) ICU C)Neurology D) others
10. The average of DNT in your hospital is around: A)<30min B)31-60min C)61-90min D)>90min
11. The percentage of your patients with AIS who received thrombolysis? A)<5% B)6-10% C)11-20%D)21-30% E)31-40% F)>40%
12. The percentage of rt-PA in your patients with AIS who received thrombolysis? A)<10% B)11-30% C)31-50% D)51-80% E)>80%
13. The most important point do you think to save time: A)timely share of pre-hospital information, B) in-hospital cooperation with other departments, C) cooperation with department of neurology, D)cooperation with high level hospitals, E) others
14. If thrombolysis has not been performed in your hospital, the reasons are: A)lack of conditions B)lack of doctors C)refused by patients D) others
15. Percentage of AIS patients need to be transferred to other hospitals: A)<5% B)6-10% C)11-20% D) 21-30% E)31-40% F)>40%
16. The time needed to transferred to other hospitals is about: A)<30min B)31-60min C)61-90min D)91-120min E)>120min
17. The most recent training you received is about: A)<1month B)1-3months C)3-6months D)6-9months E)9-12months F)>12months
18. Your title is: _______
19. How many physicians are in the department of emergency in your hospital:______
